# Behavioral Nudges to Enhance Fidelity in Telehealth Sessions (BENEFITS): Protocol for Developing and Pilot Testing a Telehealth Tool to Improve Cognitive Behavioral Therapy Implementation

**DOI:** 10.2196/76035

**Published:** 2025-09-18

**Authors:** Emily M Becker-Haimes, David S Mandell, Patty B Kuo, Kevin G Lynch, Megan Brady, Sophia Young, Torrey A Creed

**Affiliations:** 1 Department of Psychiatry University of Pennsylvania Philadelphia, PA United States

**Keywords:** telehealth, behavioral economics, implementation, cognitive behavioral therapy, evidence-based practice

## Abstract

**Background:**

The rapid expansion of telehealth provides a unique opportunity to integrate behavioral economics (BE) strategies into telehealth platforms to improve clinician fidelity to cognitive behavioral therapy (CBT)—either by enhancing clinicians’ motivation to use CBT or by helping clinicians who are already motivated to act consistently on their intentions.

**Objective:**

We will develop and evaluate “Tele-BE,” a novel telehealth platform designed to nudge and incentivize clinicians to use core structural components of CBT. We focus on these structural components because they align with practices most likely to benefit from BE strategies, are delivered across diagnoses, and represent CBT competencies independently linked to improved patient outcomes.

**Methods:**

We will refine the Tele-BE prototype in collaboration with clinicians and supervisors, who are the target end users (aim 1). Working closely with our web development team, we will field test and iteratively refine Tele-BE using rapid-cycle prototyping to optimize user experience and fine-tune the BE strategies (aim 2). The revised platform will then be evaluated in a 12-week open trial involving 30 community mental health clinicians, who will be randomized to either Tele-BE or telehealth as usual. Each clinician will deliver treatment to 2 patients, resulting in a total of 60 patient participants. All sessions will be recorded and coded to assess CBT fidelity. Clinicians and patients will complete questionnaires at weeks 1, 5, 9, and 12, with qualitative interviews conducted at the end of the trial. Primary outcomes will focus on fidelity to CBT structural components, measured via coding of recorded sessions. Secondary outcomes will include target implementation mechanisms—intentions and their determinants (attitudes, norms, and self-efficacy)—assessed using mixed methods, as well as overall CBT fidelity (aim 3). Additionally, trial data will be used to evaluate the acceptability and feasibility of Tele-BE from both patient and clinician perspectives, along with any potential ethical concerns associated with its use (aim 4).

**Results:**

The study received National Institute of Mental Health funding in June 2024. Recruitment for aim 1 began in October 2024. As of March 2025, 6 participants had been enrolled in the initial development stage. Recruitment is ongoing, and we anticipate completing aim 1 by May 2025, after which we will prepare for aim 2 activities. We aim to complete all study data collection by the end of 2026. In accordance with our grant award, deidentified data from aims 3 and 4 will be submitted to the National Institute of Mental Health Data Archive for participants who consent to data sharing.

**Conclusions:**

Findings will provide insight into the utility of a BE-informed telehealth platform for increasing clinicians’ use of core structural CBT components, thereby improving overall CBT fidelity and patient outcomes. Results will also inform the design of future confirmatory trials.

**Trial Registration:**

ClinicalTrials.gov NCT06601062; https://clinicaltrials.gov/ct2/show/NCT06601062

**International Registered Report Identifier (IRRID):**

DERR1-10.2196/76035

## Introduction

### Background

Telehealth for psychological interventions has existed for decades, with historically limited uptake, primarily due to strict regulations [1] and limited reimbursement [2]. COVID-19 facilitated the swift global adoption of telehealth [3], and payors have acknowledged the need to ease regulatory burdens and sustain telehealth reimbursement postpandemic [4-8]. To date, telehealth has primarily been studied as an alternative to in-person care [9-12] and as a means of supporting access to care for historically marginalized groups [13,14]. However, little research has examined how to leverage the platform to support evidence-based practices (EBPs) and patient-centered care.

Telehealth provides a unique opportunity to apply behavioral economics (BE) principles to structure therapy encounters in ways that nudge clinicians toward delivering high-quality services. BE approaches address psychological biases that influence individual decision-making [15]. These frameworks recognize that human decision-making often occurs under suboptimal conditions, leading individuals to deviate from “rational” choices in numerous ways. Classic examples of such “bounded rationality” (ie, the recognition that humans make decisions with limited cognitive resources and thus rely on heuristics [16]) include individuals failing to opt in to retirement investments that would serve their best interests (due to status quo or loss aversion bias [17]) or making suboptimal decisions in cognitively demanding situations (eg, as a result of decision fatigue or availability heuristics [15]). In health care, clinicians may be aware of the right course of action (eg, “I should deliver x treatment for y condition”), yet often choose other, less empirically supported interventions for various reasons related to environmental cues (eg, availability biases), individual cognitive styles (eg, aggregate heuristics), and other biases [18]. Research has shown that structuring environments and nudging clinicians toward recommended practices can improve quality of care [19-21]. Common BE applications include strategies that guide decision-making by providing information (eg, social reference points or peer comparisons, making key information visible), implementing structural changes (eg, changing choice defaults, altering options presented to influence the attractiveness of some choices over others), and offering cognitive assistance (eg, reminders) [18,22,23].

While BE holds strong potential to inform cost-effective, pragmatic implementation strategies that support clinicians in delivering EBPs, its role in changing mental health clinicians’ behavior has received little attention. The nature of traditional behavioral health sessions—occurring behind closed doors and prioritizing privacy—makes it difficult to implement supports during encounters. The continuation of telehealth access postpandemic provides a context ripe for improving treatment delivery. Telehealth has traditionally been regarded as a medium for delivering treatment, with content and processes comparable to in-office visits [24]; however, it also offers an infrastructure well-suited to BE strategies that can optimize clinician use of EBPs, track and reward fidelity to EBPs, and make EBP use easier and more motivating.

In this study, Behavioral Nudges to Enhance Fidelity in Telehealth Sessions (BENEFITS), we will develop and pilot test an implementation strategy that leverages the technological infrastructure of telehealth encounters to nudge and incentivize clinicians to increase fidelity to the structural components of cognitive behavioral therapy (CBT). We focus on CBT because it is an EBP for many mental health conditions, and increasing clinician fidelity to CBT can enhance care quality and improve patient outcomes [25-27]. Prior research suggests that when community clinicians use CBT at all, it is often delivered with poor fidelity [28-30].

We focus on structural components of CBT for 2 reasons. First, they represent a core and distinct CBT competency [31,32] and form the backbone of CBT practice; they are delivered regardless of diagnosis. A typical CBT session begins with a brief check-in on treatment progress, a collaborative agenda, and a review of prior homework. The core of the session emphasizes intervention techniques and skills, and it concludes with planning a between-session homework practice. While the specific clinical content—such as which interventions are selected and what homework is assigned—is tailored to the patient’s presentation, use of these structural components remains consistent across sessions. Structural components also support shared decision-making and facilitate the selection and tailoring of interventions to patient preferences and symptoms, thereby enhancing patient-centered care [33]. Clinical trials further suggest that these components are independently associated with improved clinical outcomes [34-37]. Our work has shown that community clinicians demonstrate consistently low fidelity to structural components [32,38], even when their intentions to deliver them are high [39].

Second, structural components are well-suited to BE strategies, which are best applied to change discrete, observable, and measurable behaviors. Leading theories of clinician behavior change [40-42], along with our prior studies [38,39], suggest 2 major reasons why clinicians trained in CBT fail to use CBT structural components—each with distinct implications for designing implementation strategies using BE approaches. First, some clinicians may have strong motivation (or intentions) to use structural components; however, aspects of their organizational setting or their patients’ presentation in treatment may make it difficult to act on those intentions. For example, a patient may present with much to share, and the clinician may forget to set the agenda or be reluctant to pause the client long enough to do so. For these clinicians, effective implementation strategies should either make it easier to act on their intentions or bypass intentions altogether by making the use of structural components the default (eg, automatically sending progress monitoring measures to clinicians rather than requiring them to do so). Second, other clinicians may have weaker intentions to use CBT components due to factors that influence intention strength, such as holding negative attitudes about a specific component (eg, believing homework is unhelpful or irrelevant), experiencing limited normative pressure to use CBT components (eg, perceiving that one’s supervisor disapproves of structured sessions), or having low self-efficacy (eg, lacking confidence in planning a homework assignment). For these clinicians, effective implementation strategies should strengthen intentions by improving attitudes, leveraging norms, or increasing self-efficacy to promote behavior change. BE offers strategies to address the mechanisms of action identified in both pathways and thereby improve clinician CBT delivery.

The field of surgery provides a useful example of the potential benefits of BE strategies for improving patient care. Surgeries are complex, requiring providers to execute highly specified procedural techniques while also knowing how and when to deviate in response to diverse problems or complications [43]. There is extensive evidence demonstrating the effectiveness of structuring surgical environments to minimize risk, optimize outcomes, and nudge surgeons to engage in safety and quality checks (eg, surgical checklists) [19,20]. Such behavioral infrastructure facilitates the delivery of high-quality care by reducing the cognitive load of remembering routine protocols [21], allowing surgeons to focus their efforts on applying clinical skill and judgment to optimally help patients. CBT structural components, when implemented correctly, provide the scaffold that supports clinicians in delivering CBT intervention techniques, which can be complex and often require adaptation [44,45] and tailoring [46] in response to patient needs that may change within and between sessions [47]. Unlike surgery, the private, in-person therapy environment has historically offered limited opportunities for BE strategies to support clinicians in delivering these structural components; therapy is typically conducted without a support team, as found in an operating room. Leveraging telehealth encounters to embed checklists and other nonintrusive nudges may reduce clinicians’ cognitive load, allowing them to focus more fully on selecting and delivering interventions and skills that yield comparable benefits for their patients.

### Goal of This Study

In BENEFITS, we will develop and evaluate Tele-BE, a novel telehealth infrastructure designed to nudge and incentivize clinicians to use core structural CBT components. Tele-BE is intended solely to support fidelity to the structural elements of CBT that should be present in sessions, regardless of where a patient is in active treatment (with expected exceptions such as first sessions, termination sessions, or crisis sessions). Tele-BE will remain agnostic to intervention content (ie, clinicians will be encouraged to use a CBT skill in session, but not any specific skill). Importantly, in other contexts, BE strategies have raised ethical concerns regarding coercion to engage in behaviors one might not otherwise choose [48,49]. Therefore, studying BE strategies to support mental health EBP delivery should incorporate careful consideration of participants’ and end users’ ethical concerns. We will address any concerns raised by clinicians or patients to ensure that clinicians do not feel undue pressure to deliver therapeutic care they consider inappropriate. Our specific aims are listed in Textbox 1.

**Study aims.**

**1. Aim 1**

Design the Tele-BE prototype to increase clinician fidelity to cognitive behavioral therapy (CBT) structural components, in collaboration with 6 clinicians and 3 supervisors (target end users), guided by user-centered design principles.

**2. Aim 2**

Adapt and iteratively refine Tele-BE in an open field trial using rapid-cycle prototyping and testing with 5-10 clinicians and 5-10 patients.

**3. Aim 3**

Test the preliminary effectiveness of Tele-BE versus telehealth as usual in engaging target mechanisms and enhancing clinician fidelity to CBT in a 12-week randomized pilot trial (n=30 clinicians, 60 patients). Primary outcomes will be clinician fidelity to CBT structural components, measured via coded therapy sessions. We will also explore mechanisms of action—including intentions and determinants of intentions (attitudes, norms, and self-efficacy)—through surveys and interviews.

**4. Aim 4**

Evaluate the acceptability, feasibility, and ethical considerations of Tele-BE from both clinician and patient perspectives using surveys, interviews, and web analytics. We will apply mixed qualitative and quantitative methods to assess Tele-BE’s impact on clinician treatment delivery, therapeutic alliance, patient satisfaction, and clinical outcomes.

## Methods

### Study Setting

All research activities will take place in the City of Philadelphia, building on a strong history of CBT implementation efforts in the region through the Beck Community Initiative (BCI). The BCI is a partnership with Philadelphia’s Department of Behavioral Health and Intellectual Disability Services to implement high-quality CBT in the public mental health system. BCI provides training in CBT theory and strategies through intensive workshops, followed by 6 months of weekly case consultation. During the consultation phase, therapists submit recorded therapy sessions, and their CBT competence is rated in at least 4 sessions using the Cognitive Therapy Rating Scale (CTRS). Mean competence scores at the end of training (41.2) have been just above the criterion threshold for certification, highlighting substantial opportunities to further improve therapist skills even after training [50]. Importantly, BCI-trained therapists demonstrated substantial variability in their intentions to use CBT structural components following training [39]. Research also shows that training effects diminish over time without additional support, such as behavioral nudges [51]. Philadelphia’s Medicaid behavioral health payor continues to support telehealth reimbursement, providing an ideal context in which to test the effects of BE strategies on CBT fidelity. All participants will complete a consent process with research team members before engaging in any study activities.

### Conceptual Framework

Table 1 presents the target mechanisms and illustrates how Tele-BE is anticipated to map onto each, guided by leading behavioral theory to (1) strengthen clinician intentions to use CBT structural components when motivation is low, and (2) support clinicians in acting on their intentions when motivation is high. Multimedia Appendix 1 presents the SPIRIT (Standard Protocol Items: Recommendations for Interventional Trials) 2013 checklist for this study.

**Table 1:** Proposed Tele-BE design features, target mechanisms, and implicated BE^a^ principles.

Tele-BE features	Target mechanism	BE principle	Present in Tele-AU^b^?
Reminder message is sent to prompt the clinician to identify skills to review, introduce, or practice before sessions, and note identified skill in the web platform.	Intentions	Availability Bias: People tend to focus on what easily comes to mind	No; the clinician is directed to the standard telehealth interface only.
Clinicians are prompted to push out brief symptom measures while the patient is waiting for the session to start every 4 weeks.	Bypassing intentions; intention-behavior gap	Changing the Default: People will engage in behaviors that are easiest and in front of them	No; to collect patient symptom data for trial purposes, patients will be contacted by research staff directly to complete measures via SMS text message or email.
Template agenda of structural components alongside the embedded video that supports clinician pacing of session by adjusting which component is highlighted (eg, agenda is highlighted until the clinician clicks to indicate that the agenda was set, at which point homework review will highlight).	Intention-behavior gap	Reducing Choice Overload: People often avoid decisions if there are too many choices	No; the clinician is directed to the standard telehealth interface only.
Checkmarks indicating component has been completed; display adjusts as goals are reached.	Attitudes	Goal Gradients: People try harder when goals are within reach	No; the clinician is directed to the standard telehealth interface only.
Gamification (eg, badges) for goals met (eg, agenda completed >90% of the time). Tele-BE will provide automated congratulatory notifications for completing CBT^c^ components and delineating them in the Tele-BE platform; exact format (eg, email vs embedded in the platform) and timing to be determined in development aims. For example, a clinician may get a notification congratulating them for preselecting a CBT skill for use 3 weeks in a row.	Attitudes	Hyperbolic Discounting: Preference for a small reward sooner over a larger reward later; Injunctive Norms: Perception of whether a behavior is approved	No
Regular feedback about elements completed from the clinical supervisor (exact timing to be refined in development). We will identify key benchmarks during our development phase to trigger a congratulatory message (if fidelity benchmark is met) or an encouraging message to increase component use (if benchmark is not met), using language refined in our P50 ALACRITY pilot trial.	Norms	Social Influence and Injunctive Norms: Perception of whether a behavior is approved	No
Downloadable summary of session activities for the clinical progress note. Information from the template agenda will be downloadable to reduce the time needed to write a session note.	Intentions and self-efficacy	Integrate Desired New Task into Existing Workflow; Make it Easy and Motivating to Use	No

^a^BE: behavioral economics.

^b^Tele-AU: telehealth as usual.

^b^CBT: cognitive behavioral therapy.

### Aim 1: Designing the Tele-BE Prototype in Partnership With Clinicians and Supervisors

**Figure 1:**
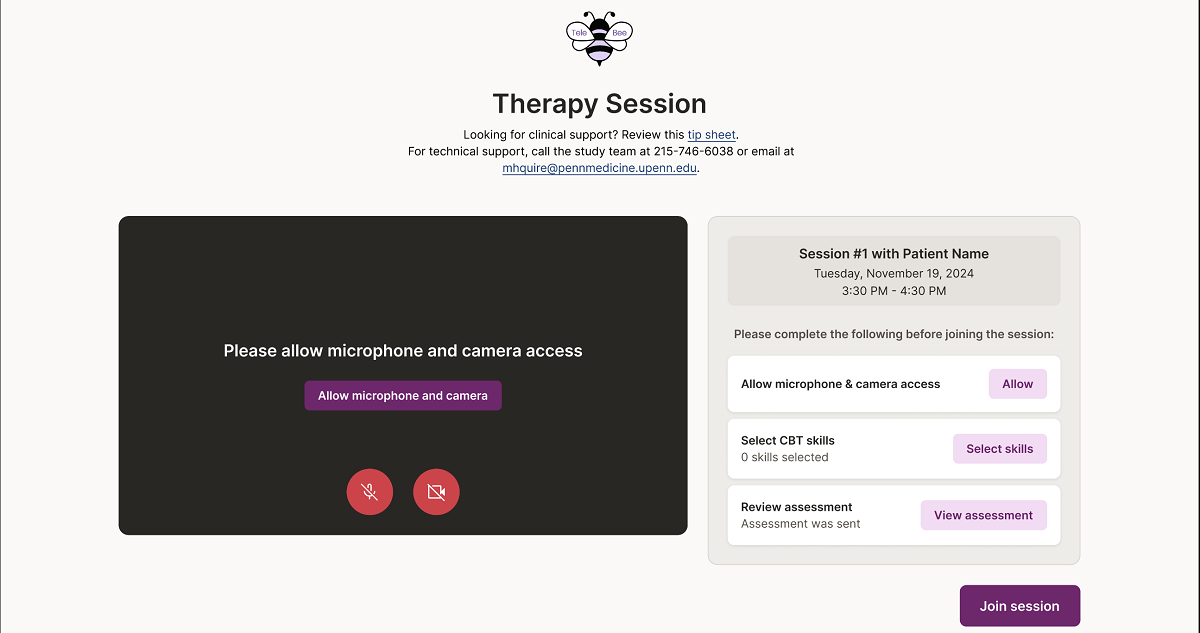
Prototyped mock-up of Tele-BE presession image. BE: behavioral economics.

Aim 1 will be conducted in 2 phases. In phase 1, we will interview 3 CBT-trained clinicians and 3 clinical supervisors who have previously received CBT training and either provide telehealth services or supervise a clinician who does. Clinicians and supervisors will not be recruited in dyads. Sample sizes are intentionally small at this stage, as the primary goal of aim 1 is to examine prototype usability to enable rapid refinement and development of the first fully functioning platform. We will present each anticipated Tele-BE feature and target mechanism (Table 1), along with initial prototypes (Figure 1), and solicit feedback on how to refine the prototype to maximize usability and engagement with target mechanisms (eg, clinician intentions, intention-behavior gap); minimize unintended negative effects on patient experience (eg, perceptions that clinicians are distracted); and ensure that Tele-BE integrates with clinical, supervision, and administrative workflows. We do not anticipate changing the structural components targeted, but we may make small adjustments to how each is described based on feedback. We will also ask whether there are additional session features that would be useful for Tele-BE to target or capture. Interviews will probe perceived motivators and barriers to adopting the Tele-BE system, including ethical concerns. For example, therapists may value the potential for improved session structure and care quality but express concern about patient perceptions of recording or about the platform infringing on their autonomy to make clinical decisions. Such feedback is critical for design and implementation planning (eg, clarifying that the interface is clinician-facing only and that clinicians can override prompts). Participants will also complete a brief demographics questionnaire to ensure that feedback is gathered from a diverse group across study phases. Recommendations from each participant will be summarized and reviewed by the larger research team to identify refinements before moving to the next phase. Results will be shared with the technology development team, which will build an initial Tele-BE working prototype incorporating this feedback. We will then reconvene the same participants for a second interview, during which they will trial the interactive prototype and suggest further refinements. All feedback will be collated and synthesized, with input echoed by multiple participants given greater weight in decisions about changes. Participants will receive US $100 in compensation for their activities, and their feedback will be integrated into the next Tele-BE iteration. Draft interview guides for aim 1, phase 1 activities are provided in Multimedia Appendix 2.

In phase 2, we will conduct usability testing with 3 newly recruited CBT-trained community clinicians, using the same eligibility criteria as in phase 1. Participants will interact 1-on-1 with a basic version of Tele-BE, with the primary objective of identifying major bugs and usability issues in preparation for aim 2. Consistent with phase 1, the small sample size is intentional, as the focus of this phase is to assess usability among clinicians, the tool’s primary end users. Each clinician will meet individually with the study team and use the current version of Tele-BE for approximately 45 minutes. During this session, participants will conduct a mock therapy session with a graduate student clinician experienced in providing community-based CBT, followed by an immediate debrief to obtain feedback on both the clinician’s end user experience and the “patient’s” perspective. Participants’ interactions with Tele-BE will be recorded using Health Insurance Portability and Accountability Act (HIPAA)–compliant videoconferencing software. Following the session, participants will complete a brief interview assessing the user experience, including learnability, acceptability, usefulness, overall satisfaction with aesthetics, perceived efficiency, and potential for integration with both clinical and supervision workflows. Semistructured interviews will probe each user interface element of the Tele-BE prototype. At the end of the session, participants will also complete brief quantitative measures to assess perceptions of Tele-BE, including the Acceptability of Intervention Measure (AIM) [52], a validated 4-item scale assessing the perceived acceptability of an innovation; the Feasibility of Intervention Measure (FIM) [52], a 4-item scale evaluating perceived feasibility; and the System Usability Scale (SUS), a well-established 10-item measure with strong psychometrics and sensitivity to change [53,54]. Participants will also complete a brief demographics questionnaire and receive US $75 for their participation. Collected data will identify areas for improvement in acceptability, feasibility, and usability, and refinements will be incorporated before aim 2.

### Aim 2: Adapt and Iteratively Refine Tele-BE in an Open Field Trial

We will recruit 5-10 clinician-patient dyads who are already providing or receiving telehealth therapy services to engage with Tele-BE in an open field trial. The final sample size will be determined based on when saturation is reached (see below). The primary aim of this trial is to identify software bugs and evaluate usability and acceptability issues that emerge over extended use of Tele-BE, while refining our BE strategies. Secondary goals are preliminarily assessing the extent to which Tele-BE engages target implementation mechanisms and supports clinicians in delivering CBT structural components to guide further refinements. Clinicians and patients will be recruited in pairs, with participating clinicians using Tele-BE with a consenting patient of their choice for up to 4 consecutive therapy sessions or over 5 weeks of treatment, whichever occurs first. After obtaining consent, each clinician-client dyad will receive a personalized, secure link to use during their regularly scheduled telehealth sessions for up to 5 weeks. Only the clinician will require instructions on using Tele-BE. Client participants will not view the Tele-BE interface, so their telehealth sessions will appear similar to standard care.

We will use rapid-cycle methods to test and refine both the platform and the BE features developed in aim 1, with the goal of optimizing messages and visual displays. Adjustments will be made after each dyad completes aim 2 activities, continuing until feedback saturation is reached and a version suitable for formal testing in aims 3 and 4 is achieved. To streamline this process, we will use “fake back ends” to test BE strategies whenever possible, avoiding time-consuming web programming for features that may require revision. For example, study staff may message clinicians directly to simulate portal-generated messages based on feedback. The exact content of messages may evolve over time in response to participant input. Using a fake back end allows small changes across enrolled dyads to be tested more efficiently.

As noted, the Tele-BE platform is designed to be agnostic to specific CBT interventions and only nudges the use of interventions within the standard CBT session structure. A key focus of this design phase is ensuring that clinicians understand how to “override” any recommendation to use a particular structural component, maintaining full autonomy over clinical decisions. Aside from supporting Tele-BE use during already scheduled CBT sessions, research staff will provide no guidance on session conduct. Recruiting from the BCI-trained pool of therapists ensures that the CBT structure aligns with routine care. The only restriction is that clinicians will not be asked to use Tele-BE during first patient encounters or termination sessions, as these sessions may not follow the standard structure that Tele-BE is designed to support.

After each dyad’s trial period, staff will interview clinicians and patients separately about their experiences. Clinician interviews will assess system usability using the same structured interview employed in aim 1, covering learnability, acceptability, usefulness, satisfaction with aesthetics, efficiency, and workflow integration. Semistructured questions will also explore clinicians’ perceptions of how Tele-BE influenced their use of CBT structural elements, whether it produced any desirable or undesirable effects on the session or therapeutic relationship, and whether they felt comfortable overriding platform recommendations. The final portion of the interview will solicit suggestions for improving Tele-BE. Brief patient interviews will focus on the patient experience, assessing whether Tele-BE is perceived to have any desirable or undesirable effects on the therapeutic relationship or patient-centered care processes (see Multimedia Appendix 3). Clinicians will receive US $75 and patients US $25 for participating in these activities. We will use rapid qualitative analysis procedures to synthesize feedback and identify changes needed for the next testing cycle. All feedback will be reviewed with the full research team to resolve any conflicting information.

### Aims 3 and 4: Evaluate the Preliminary Effectiveness of Tele-BE in Engaging Target Mechanisms and Enhancing Clinician Fidelity to CBT

#### Overview

Although aims 3 and 4 are scientifically distinct, study procedures will occur concurrently and are described together. We will randomize 30 clinicians who already provide individual CBT via telehealth for at least 2 clients to either Tele-BE or telehealth as usual (Tele-AU), with 15 clinicians per condition. Allocation will be balanced within organizations to ensure equal representation across conditions within each agency. Each participating clinician will deliver treatment to 2 patients over 12 weeks, the standard length for many CBT protocols, and clinician fidelity to CBT structural components will be tracked in each condition. While the primary goal is to evaluate the feasibility of our design and measurement approach for a fully powered trial, we will also measure patient symptoms, therapeutic alliance, and patient-centered care to explore preliminary clinical effectiveness outcomes and assess any potential concerns with Tele-BE (eg, clinician distraction or impact on therapeutic alliance) using mixed methods.

##### Participants and Procedures

We will identify at least 4 local agencies that use telehealth and have participated in system-sponsored BCI CBT training [55] as recruitment sites. A cascaded recruitment strategy, previously successful in other studies, will be employed to recruit 30 CBT-trained clinicians who treat study-eligible patients (ie, age 14 or older, consistent with the local age of consent for mental health services; already deemed appropriate for CBT; and already using telehealth as their preferred therapy modality). In this approach, clinicians will then help select 2 patients with whom they plan to use CBT and will approach those patients about participating in the study. If a clinician has more than 2 patients with whom they plan to use CBT, 2 patients will be randomly selected from the larger pool for study participation. After obtaining informed consent, clinicians will be randomized to either the Tele-BE or Tele-AU condition. Study procedures will be identical across conditions, with the only difference being that clinicians in the Tele-BE condition will use the new telehealth tool, while those in the Tele-AU condition will use the telehealth methods established by their organization. All sessions will be video-recorded using encrypted, HIPAA-compliant technology via the clinicians’ assigned telehealth platform. Clinicians and patients will complete questionnaires at 4 time points—baseline/week 1, week 5, week 9, and week 12—and participate in a qualitative interview posttrial (Table 2).

Clinicians will receive US $150 for completing study measures (US $50 for week 1 and posttrial, US $25 for weeks 5 and 9). Patients will receive US $5 per session for providing brief self-report data and allowing their sessions to be recorded, for a maximum of US $60; those completing qualitative interviews will receive an additional US $25. Clinicians and patients in both conditions will follow the same study activity schedule and complete the same assessment battery, except that participants in the Tele-BE condition will also respond to condition-specific qualitative interview questions. Compensation rates are consistent with our prior work.

**Table 2:** Trial assessment schedule for aims 3 and 4.

Outcomes		Measures	Tele-BE^a^																										Post
				1		2		3		4		5		6		7		8		9		10		11		12			
**Aim 3 primary outcomes**																													
	Structural component fidelity	CTRS^b^ [56] and web analytics	✓		✓		✓		✓		✓		✓		✓		✓		✓		✓		✓		✓		N/A^c^		
**Aim 3 secondary outcomes**																													
	Clinician intentions	Established stems [57]	✓		N/A		N/A		N/A		✓		N/A		N/A		N/A		✓		N/A		N/A		N/A		✓		
	Clinician attitudes	Established stems [57]	✓		N/A		N/A		N/A		✓		N/A		N/A		N/A		✓		N/A		N/A		N/A		✓		
	Clinician norms	Established stems [57]	✓		N/A		N/A		N/A		✓		N/A		N/A		N/A		✓		N/A		N/A		N/A		✓		
	Clinician self-efficacy	Established stems [57]	✓		N/A		N/A		N/A		✓		N/A		N/A		N/A		✓		N/A		N/A		N/A		✓		
**Aim 4 primary outcomes**																													
	Acceptability	AIM^d^ [58] and Interview	✓		N/A		N/A		N/A		✓		N/A		N/A		N/A		✓		N/A		N/A		N/A		✓		
	Feasibility	FIM^e^ [58] and web analytics	✓		N/A		N/A		N/A		✓		N/A		N/A		N/A		✓		N/A		N/A		N/A		✓		
	Usability	SUS^f^	✓		N/A		N/A		N/A		✓		N/A		N/A		N/A		✓		N/A		N/A		N/A		✓		
**Aim 4 secondary outcomes**																													
	Patient symptoms and functioning	PHQ-8^g^, GAD-7^h^, WHODAS^i^ 2.0, and DSM^j^ level 1	✓		N/A		N/A		N/A		✓		N/A		N/A		N/A		✓		N/A		N/A		✓		N/A		
	Therapeutic alliance	WAI-SF^k^ (therapist), WAI-SF (client) [59]	✓		N/A		N/A		N/A		✓		N/A		N/A		N/A		✓		N/A		N/A		✓		N/A		
**Additional constructs of interest**																													
	Contextual factors	Qualitative interview	N/A		N/A		N/A		N/A		N/A		N/A		N/A		N/A		N/A		N/A		N/A		N/A		✓		
	Clinician and patient demographics	Demographics survey	N/A		N/A		N/A		N/A		N/A		N/A		N/A		N/A		N/A		N/A		N/A		N/A		N/A		
	Supervision practices	Adapted SerTIFY^l^-Supervision Form	✓		N/A		N/A		N/A		N/A		N/A		N/A		N/A		N/A		N/A		N/A		N/A		✓		
	Clinician social desirability	Edward’s Social Desirability scale	✓		N/A		N/A		N/A		N/A		N/A		N/A		N/A		N/A		N/A		N/A		N/A		N/A		
	Tele-BE costs	Qualitative interview and web analytics	N/A		N/A		N/A		N/A		N/A		N/A		N/A		N/A		N/A		N/A		N/A		N/A		✓		

^a^Tele-AU: telehealth as usual.

^b^CTRS: Cognitive Therapy Rating Scale.

^c^N/A: not applicable.

^d^AIM: Acceptability of Intervention Measure.

^e^FIM: Feasibility of Intervention Measure.

^f^SUS: System Usability Scale.

^g^PHQ-8: 8-item Patient Health Questionnaire.

^h^GAD-7: 7-item Generalized Anxiety Disorder Scale.

^i^WHODAS: WHO Disability Assessment Schedule 2.0.

^j^DSM: Diagnostic and Statistical Manual of Mental Disorders.

^k^WAI-SF: Working Alliance Inventory—Short Form.

^l^SerTIFY: Self-reported therapist intervention fidelity for youth supervisor.

#### Quantitative Measures: Aim 3 Primary Outcomes

The primary outcome for aim 3 is clinician fidelity to structural components of CBT. We will code all recorded therapy sessions (up to 720 sessions: 60 patients × 12 sessions) for the presence of 6 structural components: (1) discussion or review of patient symptoms, (2) homework review, (3) agenda setting, (4) skill instruction or review, (5) skill practice, and (6) out-of-session practice (homework). Coding will use items from the CTRS, a gold-standard CBT fidelity measure with 11 observer-rated items, including those indexing the specific structural components of interest (homework review and planning, agenda setting, skill instruction, and skill practice) [60]. Each CTRS item is scored on a 7-point Likert scale ranging from 0 (poor) to 6 (excellent), allowing fidelity to be defined in 2 ways: (1) the presence or absence of each component, and (2) whether each component meets a competency threshold (ie, a score of 4 or higher). Our primary focus will be on the presence/absence of each component, calculated as the sum of the binary outcomes. Presence above the competency threshold will serve as a secondary outcome. The CTRS demonstrates strong internal consistency and interrater reliability [60], as well as strong interrater agreement for general competency [32]. Importantly, the CTRS includes a subscale assessing structural competency [31], in addition to scores for specific structural components and an overall competency score.

Given the volume of recorded sessions, CTRS scores will be generated for each session using the Lyssn platform. Lyssn [61] (pronounced “listen”) is a technology start-up that employs artificial intelligence (AI) to support training, supervision, and quality assurance of EBPs. Lyssn’s cloud-based platform provides the following: (1) user management and organization of sessions, clinicians, and supervisors; (2) recording, playback, and annotation of audio or video from therapy sessions; (3) speech-to-text transcription; (4) AI-generated fidelity and quality metrics; and (5) data summaries and visualizations for feedback. Session recordings are automatically transcribed using Lyssn’s speech recognition algorithms, which were trained on over 4000 sessions [62-64]. Using transformer-based deep neural networks [65], Lyssn developed AI-generated models for the 11 CTRS codes, trained on 2494 sessions with corresponding CTRS ratings within a cross-validation framework of training and test partitions [66,67]. Evaluation on a 30% test set of sessions, distinct from the training data, demonstrated strong predictive performance. For all but 1 code (*Understanding*, which is not a structural component of interest in this study), AI-generated predictions exceeded the 80% human reliability threshold. Using AI-generated metrics, the tools achieved 100% human-level reliability for total CTRS scores and demonstrated highly accurate reliability for individual items. Lyssn maintains an in-house coding team that continuously provides new validation and calibration data to monitor and sustain the performance of the AI algorithms.

While CTRS scores will serve as our primary fidelity metric, we will also examine self-reported fidelity to CBT structural components within the Tele-BE condition using web analytics data collected through the platform. As clinicians engage with Tele-BE, they will check off each structural component as it is completed in session or indicate if they intentionally override a component (Figure 1). This will provide self-reported data on structural component use for every recorded patient session in the Tele-BE condition. These data will allow comparison between self-reported use and Lyssn-generated CTRS scores, and provide insights into how and when clinicians override the Tele-BE infrastructure, informing aim 4 assessments of acceptability.

#### Quantitative Measures: Aim 3 Secondary Outcomes

Secondary outcomes are the target implementation mechanisms that align with our theorized mechanisms of action: intentions to use structural components of CBT and determinants of those intentions (Table 1). We will measure the strength of intentions separately for each structural component using established item stems from social psychology, designed to be adaptable to any behavior of interest [68]. Intentions will be assessed with 2 items on a 7-point scale, asking how willing and how likely a clinician is to use each component, with higher scores indicating stronger intentions. The 2 items will be averaged for each component. Determinants of intentions will be assessed through attitudes toward each component, measured with 4 standard semantic differential scales [40,69] (eg, 7-point scales ranging from “extremely unpleasant” to “extremely pleasant” or “extremely wise” to “extremely foolish”). Total scale scores will be computed by averaging all *z*-standardized items within each structural component, with higher scores indicating more favorable attitudes. *Norms* will be assessed using 2 standard item stems: the perception that “other people like me” will use the component, and that important others (eg, supervisors) will approve of their use, rated on a 7-point agreement scale. *Self-efficacy* will be measured with 3 statements on a 7-point scale assessing clinicians’ perceived ability to perform each component (eg, agreement with “If I really wanted to, I could use an agenda”). All items will be asked separately for each structural component and aggregated for the primary analysis, with exploratory analyses examining variation by component.

#### Quantitative Measures: Aim 4 Primary Outcomes

Primary outcomes for aim 4 include Tele-BE acceptability, feasibility, and any potential ethical concerns raised by clinicians or patients regarding the platform. Acceptability will be assessed using the AIM [52]. Feasibility will be examined through a combination of the FIM [52] and web analytics data. Tele-BE usability will be evaluated using the SUS [53]. Psychometric support for these measures has been described above under aim 1 activities. All measures will be administered to clinicians in both conditions to allow comparison of quantitative scores for acceptability and feasibility. These measures will be supplemented with clinician and patient interviews, which will also assess potential ethical concerns, including any pressure clinicians may have felt from the platform to deviate from care they believed was in the best interest of their patients.

#### Quantitative Measures: Aim 4 Secondary Outcomes

Aim 4 secondary outcomes include patient-level outcomes such as therapeutic alliance, symptoms and functioning, and treatment satisfaction. During the trial, therapeutic alliance will be assessed every 4 weeks from both the patient and clinician perspectives using parallel versions of the Working Alliance Inventory—Short Form (WAI-SF). The WAI-SF is a validated measure consisting of 12 items that ask respondents to rate their perceptions of the therapeutic relationship on a 5-point Likert scale [56]. Patient symptoms will be assessed using the Diagnostic and Statistical Manual of Mental Disorders Level 1 Cross-Cutting Symptom Adult Measure [58] (23 items rated on a 5-point scale from 0=none/not at all to 4=severe/nearly every day, covering 13 symptom domains), the 8-item Patient Health Questionnaire [57] (8 items rated on a 4-point scale from 0=not at all to 3=nearly every day, measuring depressive symptoms), and the 7-item Generalized Anxiety Disorder Scale [70] (7 items rated on a 4-point scale from 0=not at all to 3=nearly every day, measuring anxiety symptoms). These measures will be completed by patients at 4-week intervals. Patient functioning will be assessed using the WHO Disability Assessment Schedule 2.0 (WHODAS 2.0) [59], a 12-item measure rated from 1 (none) to 5 (extremely or cannot do) across 6 domains: cognition, mobility, self-care, getting along, life activities, and participation. WHODAS 2.0 will be administered at weeks 1 and 12. Questionnaires will be sent electronically to patients at the established intervals by research staff. Patient satisfaction, including any potential ethical concerns regarding their clinician’s use of Tele-BE, will be assessed qualitatively in a subsample of participants.

#### Quantitative Measures: Additional Constructs

We will additionally assess 3 quantitative measures: (1) supervisory practices, (2) clinician social desirability, and (3) the cost of Tele-BE. Clinician-reported CBT-specific supervisory practices will be measured at weeks 1 and 12 to identify potential differences between conditions. We adapted the Self-Reported Therapist Intervention Fidelity for Youth Supervision, originally a supervisor-report measure of supervisory practices, to be inclusive of clinicians working with adult clients and to specifically capture supervision related to CBT structural components. The adapted supervisory measure consists of 14 items rated on a 7-point scale, indicating the extent to which specific supervisory techniques are used (1=not at all to 7=extensively). Additionally, the 17-item Edwards Social Desirability Scale will be included in the baseline clinician survey as a potential covariate for clinician-reported engagement with the Tele-BE platform [71]. Finally, we will track the following cost metrics to inform more formal cost evaluations in future studies: (1) anticipated annual cost per clinician user for Tele-BE, including variation based on the number of patients with whom the platform is used—these estimates will be calculated in collaboration with our web development team to account for both base subscription costs and ongoing web maintenance;; (2) time required to train clinicians to use Tele-BE, which we expect to be minimal, tracked for each clinician and averaged at study completion; and (3) time clinicians spend logged into the Tele-BE interface, captured via web analytics.

#### Qualitative: Clinician Interviews (Aims 3 and 4)

All clinicians, regardless of condition, will complete interviews after their patients finish the trial period. The first portion of the interview will be identical for all clinicians and will explore perceptions of their intentions and the determinants of intentions to use CBT structural components throughout the trial. This portion addresses aim 3 questions regarding engagement of target mechanisms. Questions will be adapted from an established theory-guided qualitative interview [72], which includes items on attitudes toward an EBP, self-efficacy, perceived norms, and motivation. Questions will be asked separately for each structural CBT component of interest (Table 1). All clinicians will also be asked about barriers they encountered during the trial in using structural components, with specific probes regarding the impact of cognitive burden on delivery (eg, “To what extent, if at all, did factors like forgetting due to competing demands make it easier or harder for you to set an agenda?”). Collecting these responses from all clinicians will allow us to examine themes by condition to determine whether Tele-BE better engaged target mechanisms and reduced barriers to structural component delivery. The second portion of the interview will be administered only to clinicians randomized to Tele-BE and will address aim 4 by querying acceptability and any feasibility issues that arose. Patient interview questions may include, “How was your experience with Tele-BE? What aspects were easy or difficult to use?” We will also use self-reported fidelity data collected via web analytics to ask clinicians specific questions about how they decided to act on or override Tele-BE nudges (eg, electing not to review a planned homework) and their rationale for doing so. Clinicians will also be asked about any ethical concerns they or their patients might have, such as undue pressure to deliver structural CBT components or potential negative impacts on treatment processes. Finally, clinicians will be queried about any unanticipated resources required to engage with Tele-BE and asked to estimate how the platform influenced the time needed to prepare for or document a therapy session, informing our cost estimates.

#### Qualitative: Patient Interviews (Aim 4)

We will randomly select 1 patient per therapist to participate in a qualitative interview at the end of the 12-week trial period. If a patient declines, the second patient per therapist will be selected, ensuring 1 patient per therapist completes an interview. In the first half of each interview, research staff will ask patients about their perceptions of the therapeutic relationship during the trial (including any changes from before the trial), satisfaction with services received over the preceding 12 weeks, and perceived effectiveness of psychosocial treatment. This will allow comparison by condition to examine whether themes related to treatment processes differ depending on whether the clinician used Tele-BE or Tele-AU. In the second half of the interview, staff will query patients about their awareness of and perceptions regarding Tele-BE or Tele-AU and its influence on clinician behavior. Staff will then provide patients with information about Tele-BE and ask about their perceived likes and dislikes regarding their clinician’s use of an enhanced telehealth interface to deliver care.

#### Preliminary Analyses

Data screening and analysis of missing data will be conducted following best practices. We will evaluate the psychometric properties of all scales used to assess constructs of interest (eg, coefficient alpha) to confirm adequate performance. Baseline variables will be analyzed as a randomization check to ensure comparable characteristics between clinicians in the Tele-BE and Tele-AU groups. We will also assess the sensitivity of our main comparisons to medium-to-large effect size imbalances between the randomized groups.

#### Quantitative Analyses

Aim 3 primary analyses will focus on structural component scores from the CTRS generated via the Lyssn platform, dichotomized as present or absent. An aggregate fidelity metric will be calculated for each session by summing the number of components present. We will use a linear mixed effects regression model to estimate the trajectory of completion scores over the 12 weeks of treatment. Fixed effects will include linear and quadratic time trends to capture the overall trajectory, although more flexible specifications will be considered if needed. To account for correlations arising from patients nested within clinicians, a random intercept for clinicians will be included, along with correlated residuals to address within-patient correlations over time. The model will use data from all 12 sessions to estimate time trends, as well as session means and standard errors for each session. Additionally, monthly estimates will be generated to align with participant survey intervals. As described above, we will also create an overall structural component sum score for each session by summing the total structural components rated as “completed at or above the satisfactory threshold” on the CTRS. If sufficient variability exists, we will model the time course using mixed effects models as described previously. If the score range is too narrow, we will use mixed effects ordinal regression models, with fixed and random effects specified similarly. All analyses will include patient demographics (eg, age, diagnosis) as covariates. In addition to CTRS-based fidelity measures, self-reported fidelity will be captured for the intervention group using web analytics data. The primary self-report measure will parallel the CTRS, summing the number of structural components rated as present. We will use a bivariate-response mixed effects model, treating CTRS and web-based measures as concurrent responses across the 12 weeks, to compare overall levels and trends over time between the 2 measurement methods.

To examine secondary outcomes (intentions and determinants of intentions), we will model monthly trends in clinician-reported intentions, attitudes, self-efficacy, and perceived social norms using approaches analogous to those described for fidelity measures. The primary distinction is that time will be treated as a categorical variable, reflecting the 3 assessment points rather than the 12 weekly sessions.

#### Missing Data Considerations

Based on ongoing prospective community mental health studies with comparable follow-up periods [73], we anticipate a clinician dropout rate of approximately 20% over 12 weeks, with minimal patient dropout among retained clinicians since patients are drawn from established caseloads. The mixed effects models described above will provide valid estimates and standard errors under the assumption that dropout is ignorable (ie, dependent only on observed outcomes and participant characteristics). We will conduct additional analyses to assess the sensitivity of our results to this ignorability assumption. Given the study’s sample size, our primary focus will be on overall completion of the 12-week protocol. We will use logistic regression models to examine whether prior outcomes or baseline variables are individually associated with study completion. Variables showing strong associations with completion will be combined into a multiple logistic regression model to predict completion. Based on participant-level dropout probabilities estimated from this model, we will perform exploratory inverse probability–weighted versions of the main mixed effects models and compare intervention effect estimates from the weighted and unweighted models.

#### Qualitative and Mixed Methods Analyses

All interviews will be audio-recorded and transcribed for thematic analysis to complement quantitative data, allowing examination of target mechanism engagement (aim 3), Tele-BE acceptability and feasibility, and potential ethical concerns (aim 4). Given our interest in understanding clinician and patient experiences separately, interviews from these groups will be analyzed independently. Qualitative coding for both clinician and patient interviews will proceed in 6 stages. First, the coding team will familiarize themselves with the data by reading all transcripts and noting initial reactions. Team members will then debrief and share observations after reviewing all transcripts. Second, the coding team will develop an initial set of codes through line-by-line open coding of 2 randomly selected transcripts. Each team member will code the transcripts independently, then reconvene to compare codes and reach consensus. This consensus coding process enhances trustworthiness in data analysis and helps account for individual values and biases that may influence coding [74]. While reviewing the transcripts, the team will create a codebook documenting code descriptions to facilitate organization and reflection. The codebook will likely be guided by a combination of a priori areas of interest (eg, Tele-BE’s impact on motivation to use structural components, acceptability of Tele-BE, and its effect on reducing cognitive burden in CBT delivery) as well as emergent themes arising directly from the data. Following codebook creation, the coding team will independently code all remaining transcripts line-by-line, compare codes, and reach consensus on coding decisions. The codebook will be iteratively updated as new codes emerge, capturing additional clinician and patient experiences revealed in the interviews. Third, the team will cluster codes based on shared patterns in content and generate themes that describe these patterns. Fourth, themes will be compared against quotes from the coded data to ensure they accurately reflect participant experiences. The team will refine themes by consolidating similar themes and breaking broader themes into subthemes as needed. Fifth, the team will finalize names and descriptions for all themes and subthemes. Finally, findings will be presented using illustrative examples from the coded data. To further enhance trustworthiness, coding team members will maintain analytic memos documenting their thought processes regarding codes, subthemes, and themes, supporting reflection on how values and biases—particularly related to Tele-BE—may have influenced coding decisions.

We will then conduct mixed methods analyses following best-practice recommendations to contextualize and interpret our quantitative findings [75-77]. Specifically, we will compare qualitative themes among therapists with low, medium, and high fidelity in Tele-BE, focusing on instances of overriding Tele-BE nudges, perceived ease of use, and any ethical concerns. Additionally, we will examine how patient ratings of therapeutic alliance are informed by their qualitative experiences with therapists using Tele-BE, and explore differences in patient-reported themes among therapists with varying levels of fidelity to structural CBT components.

#### Progression Criteria

We will examine acceptability and feasibility data, as well as trends in observed fidelity, to determine whether the pilot trial results support progression to a larger, confirmatory trial. For acceptability and feasibility, we will descriptively compare mean AIM and FIM scores between Tele-BE and Tele-AU clinicians; comparable scores would support the utility of progressing. If Tele-BE scores are notably lower than those in the Tele-AU condition, this would raise concerns regarding its acceptability and feasibility. Regarding fidelity, we will look for trends indicating (1) greater use of structural components in the Tele-BE condition relative to Tele-AU as measured by the CTRS, or (2) increased motivation to use CBT structural components in Tele-BE relative to Tele-AU, as suggested by qualitative analysis.

### Ethical Considerations

All study activities will be overseen by the City of Philadelphia (protocol number 2024-58) and the University of Pennsylvania (protocol number 855355) Institutional Review Boards (IRBs). All participants will complete informed consent before participation. Identifiable data will be stored and analyzed using HIPAA-compliant, password-protected electronic systems. Participants will receive compensation for study activities as outlined above; these rates are consistent with prior work and designed to provide appropriate incentives while minimizing the risk of coercion. To address ethical considerations related to BE strategies—specifically the potential for nudges to influence participants to engage in behaviors they might not otherwise choose—we will collect data on any concerns raised by clinicians or patients. This will ensure that clinicians do not feel coerced to deliver care they deem inappropriate, and the feedback will inform future iterations of Tele-BE to prevent undue pressure in clinical decision-making.

Any modifications to the strategy or outreach materials will be submitted to the IRBs according to each institution’s procedures. Any deviations from the protocol or adverse events will be documented and reported. All participants will receive a copy of their consent documentation before engaging in any study activities.

### Data Safety and Monitoring

We will convene a 3-member Data and Safety Monitoring Board (DSMB) composed of independent experts with no professional or financial conflicts of interest related to the study or investigators. The DSMB will be responsible for reviewing study data for quality and integrity, ensuring adherence to the protocol, monitoring participant safety, evaluating study conduct and progress, and making recommendations regarding study continuation, modifications, or suspension/termination. The DSMB will meet before patient enrollment (ie, before aim 2) and annually thereafter for the duration of the study.

## Results

The study received National Institute of Mental Health (NIMH) funding in June 2024. Recruitment for aim 1 began in October 2024. As of March 2025, 6 participants have been enrolled in the initial development stage. Recruitment is ongoing, and we anticipate completing aim 1 by July 2025, after which we will prepare for aim 2 activities. We aim to complete all data collection by the end of 2026. In accordance with our grant terms, deidentified data collected in aims 3 and 4 will be submitted to the NIMH Data Archive for participants who consent to data sharing.

## Discussion

### Contribution to Existing Work

The rapid expansion of telehealth [3] presents novel opportunities to address the therapy environment’s infrastructure through technology, an approach previously limited by the closed-door, solitary nature of traditional therapy. This pilot randomized hybrid effectiveness-implementation trial aims to develop and evaluate the initial acceptability and feasibility of a novel telehealth tool designed to improve clinician fidelity to CBT structural components. While telehealth has long been promoted as a means of increasing patient access to CBT by expanding the pool of available providers, its potential to enhance clinicians’ delivery of EBPs has not yet been fully leveraged. To our knowledge, this study represents the first effort to leverage telehealth platform infrastructure to enhance clinician delivery of EBPs, rather than treating telehealth primarily as a medium that replicates traditional face-to-face therapy. It is also among the first to apply and evaluate BE strategies for a psychosocial EBP. Our focus on the cross-cutting, transdiagnostic structural elements of CBT—rather than diagnosis-specific protocols or intervention techniques (eg, exposure therapy)—is a further point of distinction. BE strategies are particularly well-suited for changing discrete provider behaviors that are observable and measurable [15,78], and CBT structural components (eg, agenda setting) share these characteristics. This approach enables us to directly target clinician behaviors through Tele-BE in a manner that aligns with BE-informed implementation strategies. It also lays the groundwork for future research that could layer intervention-specific implementation strategies on top of those targeting structural components.

### Design Considerations

Initially, we considered linking our trial phases to sessions attended rather than calendar weeks; however, most outpatient sessions occur weekly, and no-show rates are lower with telehealth [79]. These no-show rates will inform the design of future trials. We also considered including a broader set of EBPs with structural components (eg, dialectical behavior therapy), but focused on CBT due to its implementation within our partner public health system and to maintain specificity in our patient population. Other EBPs will be examined in future work. While we considered including supervisors in aims 2-4, we prioritized initial testing with direct end users—therapists and patients. Supervisor representation is incorporated in aim 1 design, and clinicians report on supervisory practices during the trial; follow-up trials will more formally include supervisors. We also considered limiting inclusion criteria to specific patient diagnoses for our clinical trial; however, given our focus on transdiagnostic CBT, we determined this approach would be more appropriate for subsequent trials. Additionally, we considered formally measuring organizational constructs (eg, culture and climate) in this pilot; however, our primary focus is on addressing target mechanisms of action through the BE strategies. Ultimately, we anticipate that Tele-BE will be implemented as part of broader organizational quality improvement efforts, making these organizational constructs critical to evaluate in larger, confirmatory trials.

### Limitations and Efforts to Overcome Them Through Study Design

Several limitations of this study should be noted. First, although the study team aims to make Tele-BE as generalizable as possible, this pilot will not fully evaluate how it will perform across community agencies, which vary widely in resources and emphasis on telehealth service delivery [80,81]. For example, in Philadelphia, community mental health agencies differed in their capacity to transition quickly to remote care at the start of the pandemic, largely due to the costs of obtaining a HIPAA-compliant video service. To mitigate this limitation, our platform is designed to be embedded in a HIPAA-compliant web page rather than requiring a dedicated app installation, meaning clinicians will only need access to an internet browser to use it. In addition, we restrict eligibility to English-speaking patients, as our primary focus is on establishing the feasibility, acceptability, and preliminary effectiveness of Tele-BE. This restriction limits the generalizability of our findings. Finally, the 12-week duration of our pilot trial does not allow us to examine the potential for Tele-BE to support sustained fidelity over time. To address this, we will include specific questions in our aim 3 qualitative interviews with both clinicians and patients about their perceptions of using or receiving Tele-BE over a longer period, which will inform future studies on the sustainability of its effects.

### Future Directions

In addition to next steps that include a fully powered evaluation of Tele-BE, future studies will extend its use to other EBPs that employ structural components and examine the durability of BE strategies (ie, whether effects persist over time), the generalizability of effects (eg, whether increases in fidelity transfer to in-person sessions conducted by the same clinicians and whether Tele-BE ultimately improves patient outcomes), and strategies for scaling up Tele-BE if it proves effective. This work will also lay the groundwork for future research to determine whether targeting structural components, rather than CBT broadly, is a more efficient way to improve fidelity across CBT protocols and, eventually, other EBPs that include structural components, such as dialectical behavior therapy. Such work also has the potential to advance the CBT and implementation science fields by enabling disaggregation of treatment effects associated with structural components from intervention technique fidelity. We will also explore additional strategies to leverage the telehealth infrastructure to make it easier and more motivating for clinicians to deliver evidence-based care, such as embedding links to CBT worksheets or tip sheets/session guides for common CBT skills delivered during sessions. Of note, Tele-BE is intended to address some, but not all, of the barriers clinicians face in delivering CBT. The nudge approach proposed here represents a low-cost, low-barrier method with substantial potential to increase clinician fidelity to a level that could meaningfully improve clinical outcomes.

### Conclusions

This study will provide insight into the potential of leveraging the telehealth infrastructure to improve CBT fidelity by integrating BE strategies into the telehealth interface. If the study produces promising results, it will pave the way for large-scale testing of Tele-BE to enhance clinician CBT fidelity and patient outcomes across mental health settings that provide telehealth services.

## Appendix

Multimedia Appendix 1 SPIRIT 2013 checklist.

Multimedia Appendix 2 Draft interview guides for aim 1 activities.

Multimedia Appendix 3 Draft of aim 2 interview guides.

## Abbreviations

AI: artificial intelligence
AIM: Acceptability of Intervention Measure
BCI: Beck Community Initiative
BE: behavioral economics
BENEFITS: Behavioral Nudges to Enhance Fidelity in Telehealth Sessions
CBT: cognitive behavioral therapy
CTRS: Cognitive Therapy Rating Scale
DSMB: Data and Safety Monitoring Board
EBP: evidence-based practice
FIM: Feasibility of Intervention Measure
HIPAA: Health Insurance Portability and Accountability Act
IRB: Institutional Review Board
NIMH: National Institute of Mental Health
SPIRIT: Standard Protocol Items: Recommendations for Interventional Trials
SUS: System Usability Scale
Tele-AU: telehealth as usual
WAI-SF: Working Alliance Inventory—Short Form
WHODAS: WHO Disability Assessment Schedule 2.0
