# Supplementary material for: Behavioral Nudges to Enhance Fidelity in Telehealth Sessions (BENEFITS): Protocol for Developing and Pilot Testing a Telehealth Tool to Improve Cognitive Behavioral Therapy Implementation
Source: JMIR Res Protoc. 2025 Sep 18;14:e76035. doi: 10.2196/76035 (PMC12491885; doi:10.2196/76035)
Supplement: Multimedia Appendix 2 [file resprot_v14i1e76035_app2.docx]

**Aim 1, Stage 1:**  **Semi-Structured Interview Guide**

*Note that this interview is intended to be a guide only –facilitator should ask some or all of these questions depending on what information is shared by participants.*

**Introduction**

***Thank you all for your study participation. To learn more about your thoughts on the new tool we have developed, we will record our discussion and keep everything you share fully confidential, as allowable by law. Your responses will not be shared with your organization.***

***There are no right or wrong answers; we want to hear your perspectives so we can continue to improve and make our new tool as maximally helpful as possible. You can choose not to answer any questions, or you can leave the discussion at any time.***

***As a reminder, we ask that you respect the privacy of others in the group and do not share personal or identifying information about anyone here outside of this setting. We also ask that we all make efforts not to interrupt one another when speaking and aim to respect others’ opinions throughout our time together.***

***Do you have any questions before we begin?***

**[RECORD]**

1. **[Orient to Tele-BE]. *Our goal is to help clinicians be able to deliver CBT more effectively. CBT is a complex intervention and clinicians are asked to do a number of things within a general structure in each session. We are trying to build a telehealth tool to help clinicians follow this structure in session more easily. The tool will include reminders and tips for using specific elements of CBT.***
2. ***I want to show you an initial version of this tool, which we are calling “Tele-BE” for now. After we give you the big picture, we’ll go back through each component to ask for your thoughts about them.***

***[screenshare]***

**Pre-session pages**

***
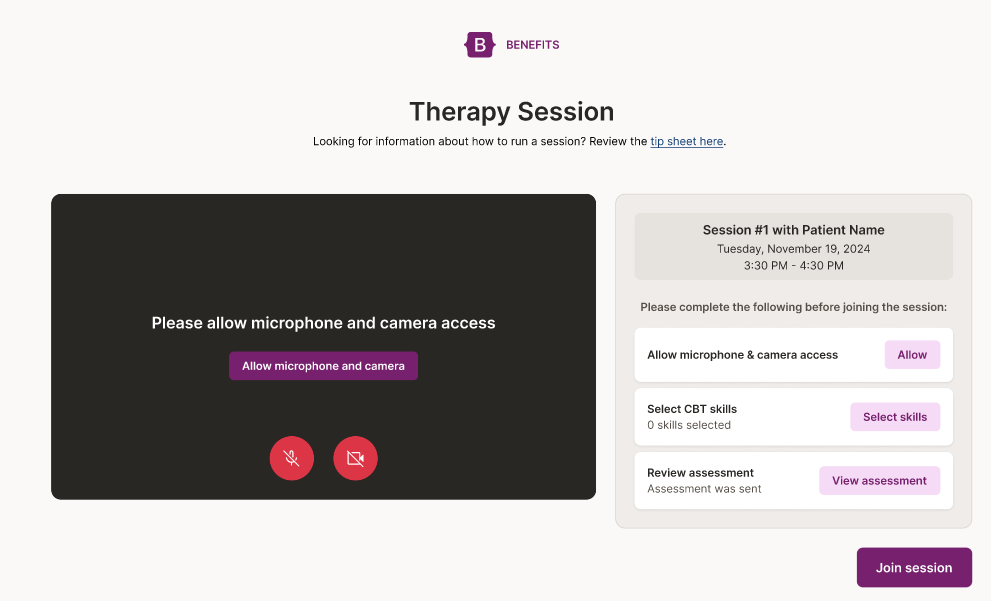
***

***
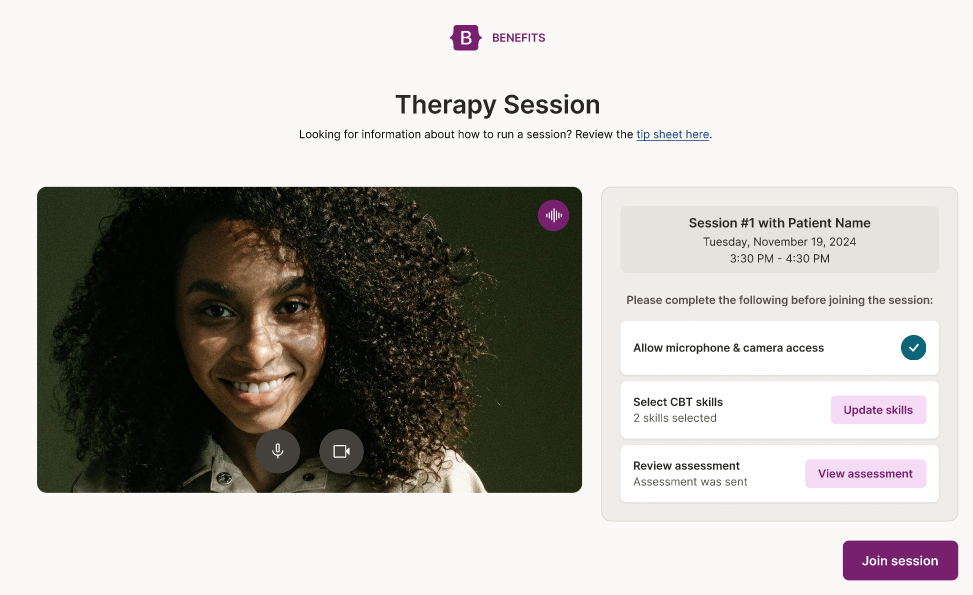
***

- ***Here is our first pass of what this tool could look like – this is what your screen as the clinician would look like. Your client’s screen will NOT have this display and will look like a regular videochat.***
- ***The center screen will display your video chat with your client like it normally would. The functions will remain the same as a typical video chat.***
- ***On the right-hand side, you can see what looks like a checklist side-bar. The goal of this feature is to help clinicians set and maintain their session agenda and follow the typical structure of a CBT session***
- ***It is designed to be interactive so that as you go through your session, you can check off each part of your session structural component as you complete it with your client. However, the goal is to make SUGGESTIONS only and not to require things. You may feel a specific part of session structure isn’t a good fit for your client – if so, you would click “CHOSE TO SKIP” to indicate that you decided not to do that part on purpose.***

***I’d love to hear your initial thoughts on this tool – tell me your initial reactions***

***[SHOW PRE-SESSION PAGE: PAUSE FOR FEEDBACK- PROBE FOR GENERAL FEEDBACK ON LIKES, DISLIKES, PERCEIVED UTILITY]***

**In session pages:**

***
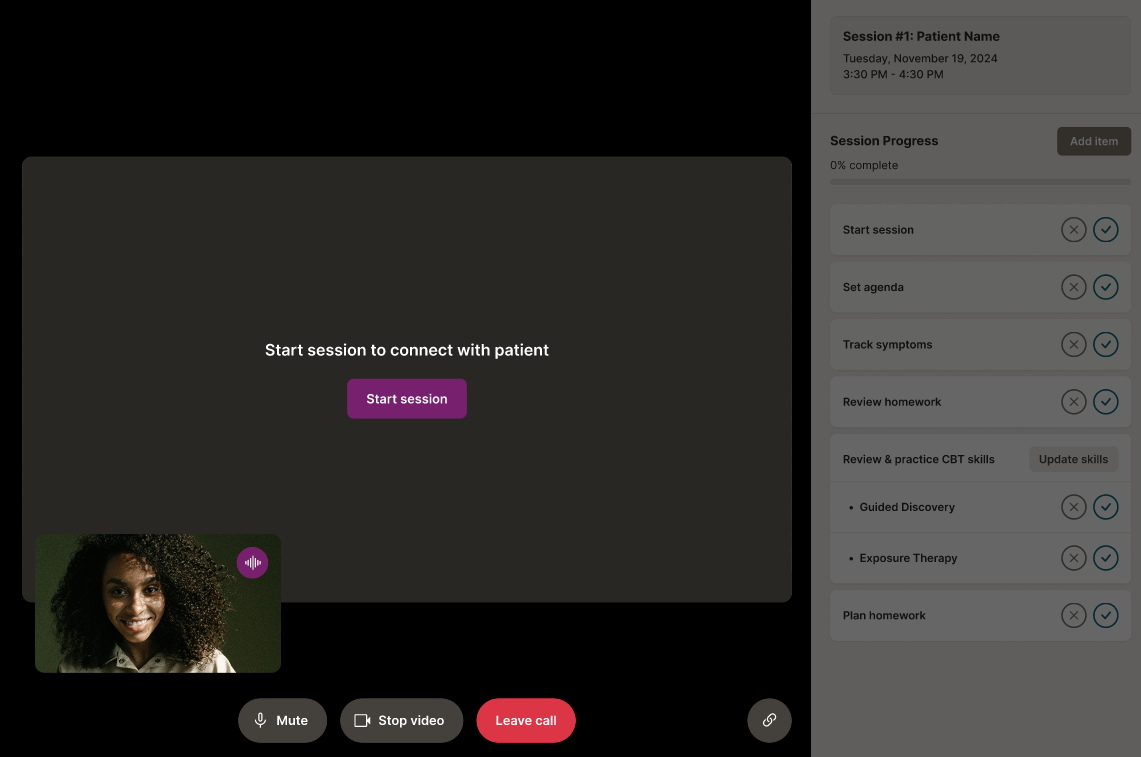
***

***
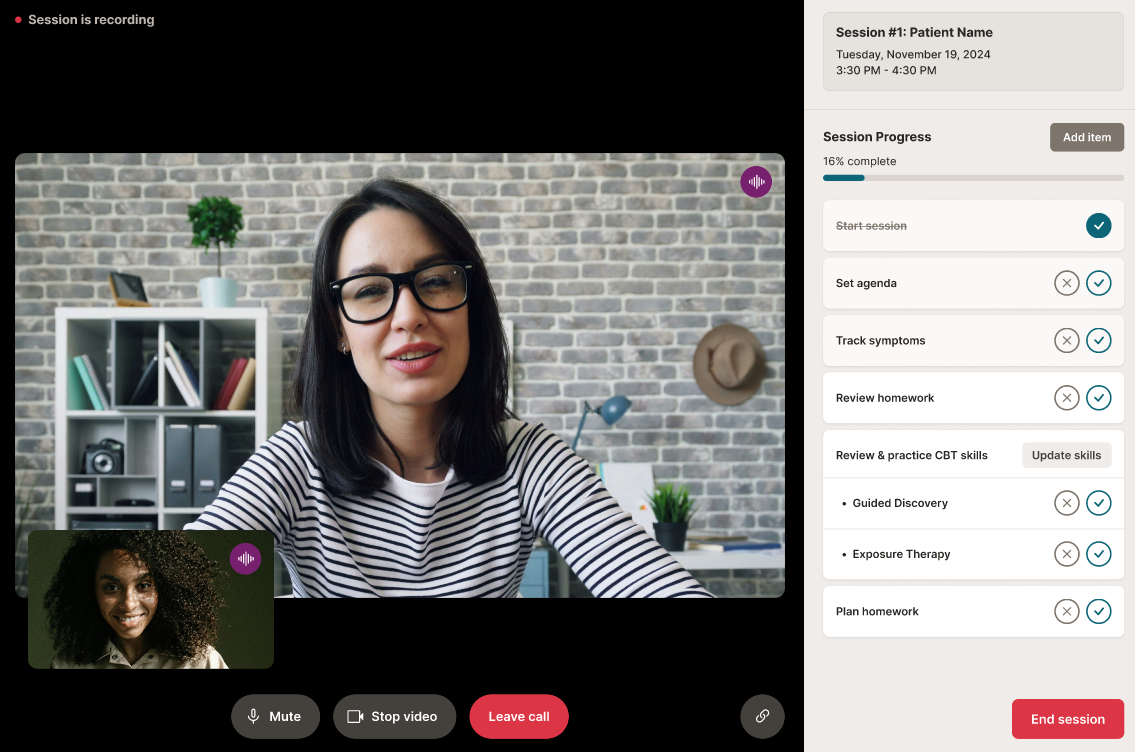
***

- ***In addition to what you can see here, we also plan to build in messages to cheer for achievements and pre-session reminder prompts. Let me show you what that initial screen would look like***
  - ***This is what you would see first when you go to join your client session. First, the screen will prompt you to identify what CBT skill or intervention you think you are most likely to use in session with this client – you will see there is also an option to click “unsure/depends on client needs”***
  - ***Finally, there will be an option here to send brief survey measures to your client about their symptoms (anxiety, depression) and how they feel about the therapeutic alliance. You would receive scores on those measures to inform treatment planning***
  - ***Probe:***
    - ***You can see here that there is a checklist, are there things on this list that you regularly do as part of your session structure that is not included on this checklist? Is there anything here where you would use different language to describe this?***

***[PAUSE FOR FEEDBACK- PROBE FOR GENERAL FEEDBACK ON LIKES, DISLIKES, PERCEIVED UTILITY]***

**Here is what your client will see during your session:**

**Client screen**

***
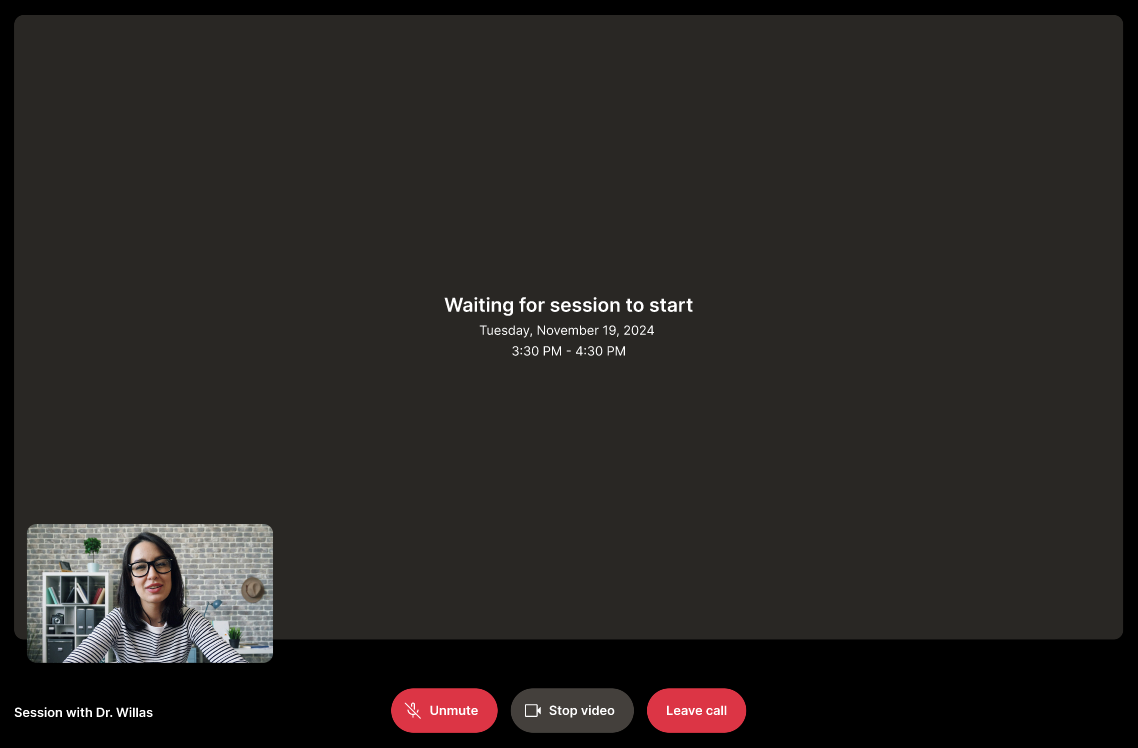
***

***
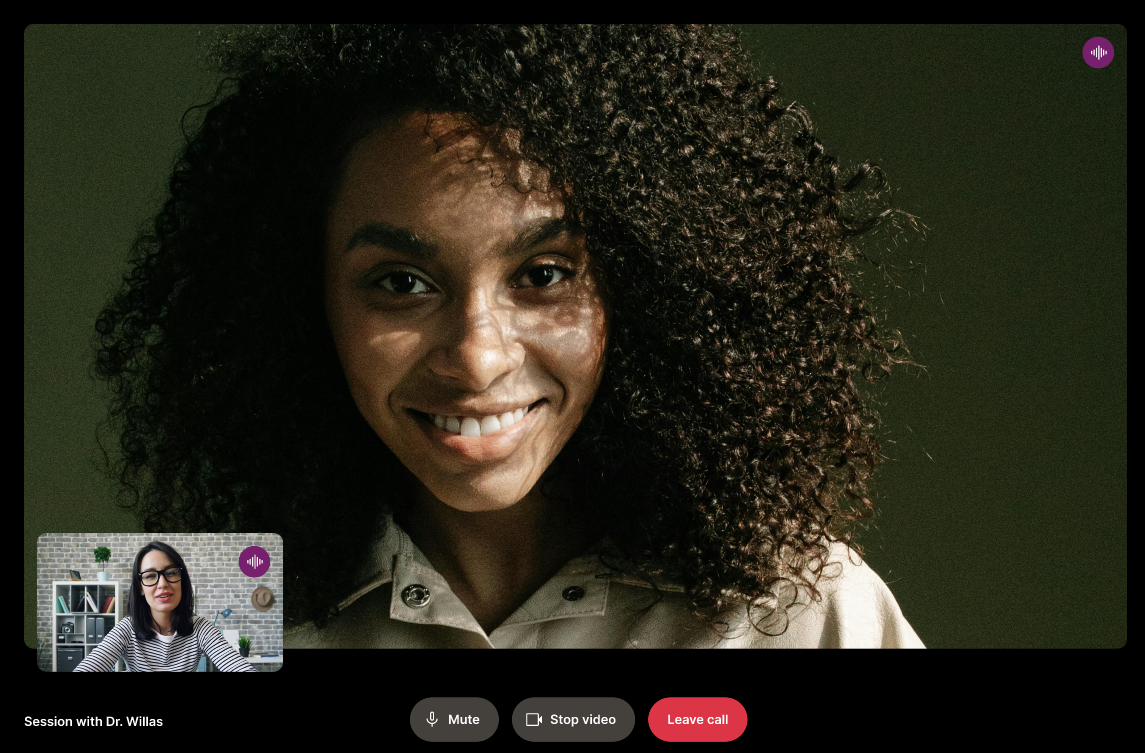
***

***We also want to get your more specific thoughts on each of the planned Tele-BE features. First though, is there any other big picture feedback on what you like and dislike about this tool?***

***Next, I would like to get your thoughts on each planned feature of Tele-BE. To***

1. ***As we just discussed, when you’re ready to enter the virtual therapy room, you would be prompted to identify a skill or skills to review, introduce, or practice. The identified skill(s) will be incorporated into the checklist after entering session.***
   1. ***How helpful or unhelpful would this be for you in the moment?***
   2. ***Probe for additional information.***

***Once you join the session, you would see the session CBT checklist next to the video. Checklist items would be: (1) review last session, (2) set agenda, (3) measures and mood check, (4) discuss past homework, (5) teach, review, or practice skill, and (6) plan homework. Within skill discussion, you would also check off if you introduced, reviewed, or practiced a skill in session as well as check off the skill used with the same checklist you saw before entering the therapy room***

***As you complete each item on the checklist, you would check them off in real time in the session - then the item would have a checkmark or turn green to show it was completed or intentionally skipped.***

- 1. ***How helpful or unhelpful would this be for you?***
  2. ***How do you feel about the order of the items on the checklist?***
  3. ***Probe for additional information.***

1. ***It’s important to us that clinicians feel like they can easily click “N/A” for each option. For example, if you didn’t assign a HW session the prior week, there would be no need for homework review in this session. How clear/easy does it seem like it would be for you to indicate that you chose intentionally not to do something on the checklist because of your client’s needs?***
   1. ***What could we do to make it easier or clearer about how to indicate a session item was N/A?***
2. ***At the end of session, a downloadable summary of session activities based on the checklist would be available to copy from Tele-BE and paste into the “Action” section of the clinical progress note. For example, if someone completed all elements, it might read something like:***

***Clinician administered progress monitoring tools, set agenda, checked in on treatment progress, reviewed homework, reviewed CBT skill (cognitive restructuring), and planned for home practice.***

- 1. ***How helpful or unhelpful would this be for you?***
  2. ***Probe for additional information.***
  3. ***What if any potential organizational barriers might there be to this feature (e.g., some programs do not allow copy/paste from external systems)?***

***
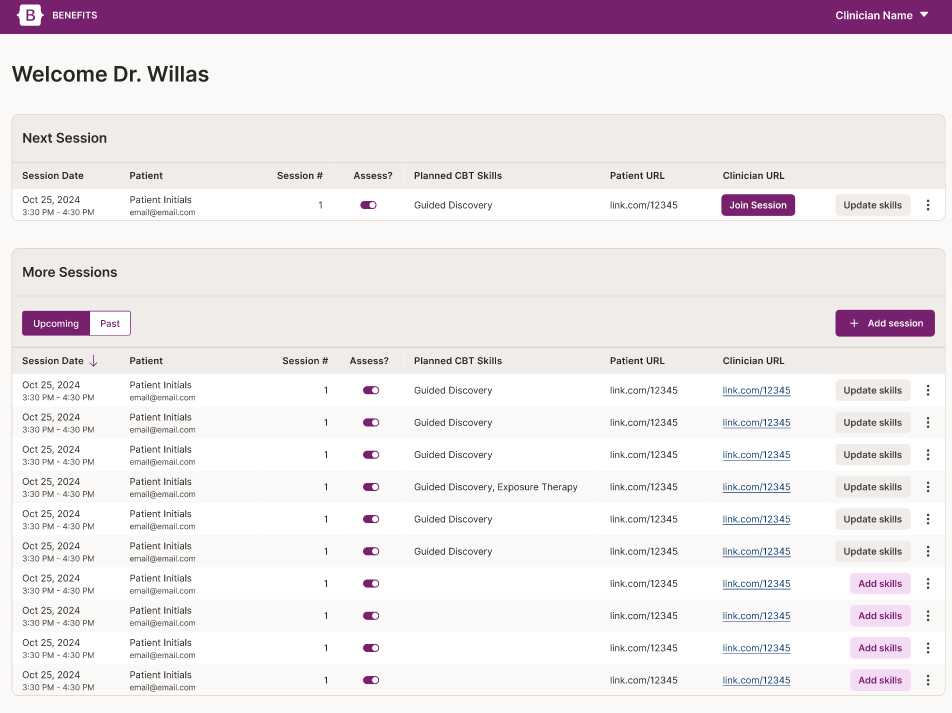
***

***
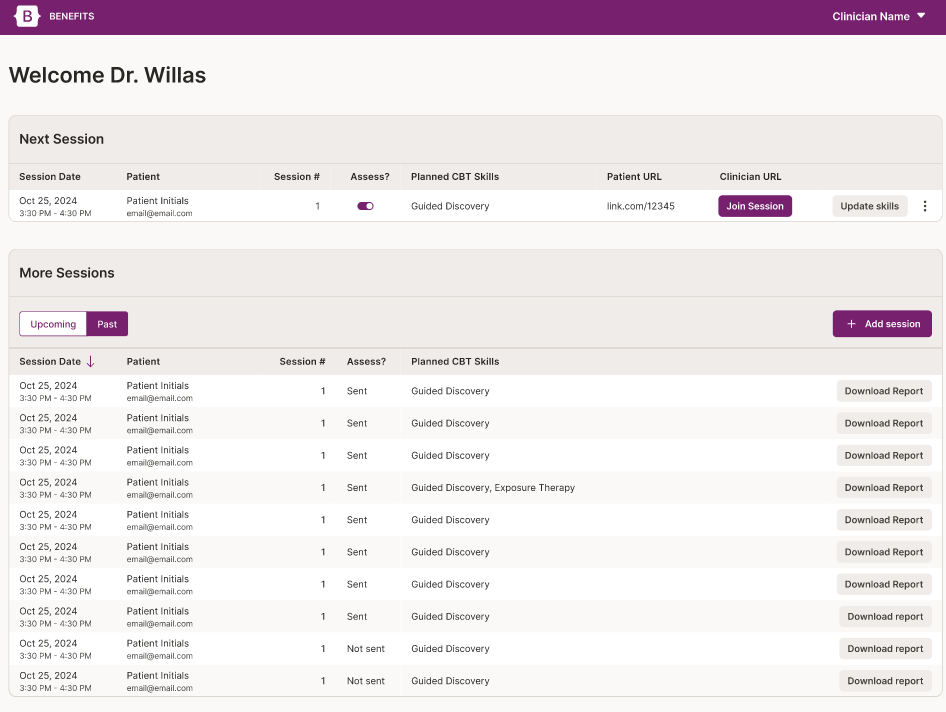
***

1. ***FOR CLINICIANS ONLY: After the session, we plan for the platform to provide automated messages congratulating clinicians for using CBT components and marking them off them in the Tele-BE platform. For example, a clinician may get a notification congratulating them for planning to use a CBT skill in session for three weeks in a row. Another example might be “Congratulations – you set an agenda >90% of the time!”. For session checklist items that are used less often, clinicians also get message along with a link to a tip sheet to support its use. For example, “You haven’t set an agenda with your clients in the last few sessions. There are lots of reasons why you might choose not to set an agenda with a client, and other times you might find that info about agendas is useful. Click HERE for some tips on how and when to set an agenda in session”***
   1. ***How helpful or unhelpful would this be for you?***
   2. ***Probe for additional information.***

***How would it be most helpful for you to receive this information? For example, would you rather receive it in an email or embedded in the platform?***

- 1. ***When would be most helpful to receive this information (e.g., immediately after session, in an email a day after session, etc.).***

1. ***FOR SUPERVISORS ONLY: After the session, we plan for the platform to provide automated messages congratulating clinicians for using CBT components and marking them off in the Tele-BE platform. For example, a clinician may get a notification congratulating them planning to use a CBT skill in session for three weeks in a row. Another example might be “Congratulations – you set an agenda >90% of the time!”. For session checklist items that are used less often, clinicians also get message along with a link to a tip sheet to support its use. For example, “You haven’t set an agenda with your clients in the last few sessions. There are lots of reasons why you might choose not to set an agenda with a client, and other times you might find that info about agenda is useful. Click HERE for some tips on how and when to set an agenda in session”***

***We know that there are many reasons a clinician may not use all CBT checklist items in a single session. We want to identify what the best benchmarks are that should trigger a congratulatory or encouraging message. For example, in what percent of sessions would you expect a CBT clinician to set an agenda, knowing that things often come up that may lead a clinician not to do so for clinically appropriate reasons, such as a client coming in with a crisis?***

***How often would you want them to be checking in on treatment progress?***

***What benchmark would you ideally see for how often clinicians should reviewer or set homework?***

***What about introducing, reviewing, or practicing a CBT skill?***

1. ***Now let’s talk about the design of Tele-BE [re-show image]. This is what you will see when you log into a telehealth encounter. The patient’s view will remain unchanged and they will not see these components. Tell me your initial thoughts about what you like and dislike about what you see.***
   1. ***Probe specific features if not discussed:***
      1. ***Color***
      2. ***Font***
      3. ***Arrangement***
   2. ***How easy or hard does this seem like it would be to navigate? How can we make it easier to use?***
   3. ***What instructions do you think would be needed to help you use this tool easily?***
   4. ***The current version is only developed for laptop or computer use. Would you ever have a need to conduct a therapy session from your phone?***
2. ***Now that you’ve seen the details of the tool, do you have any feedback about the name ‘Tele-*BE’?**
   1. ***Probe if necessary:* *Do you have any recommendations about the name?***
3. ***We want to make Tele-BE as easy to use as possible. How do you see something like Tele-BE fitting into your clinical workflows?***

***Thank you so much for your time. We really appreciate all of this feedback. Is there anything else you would like to share with us?***

***Provide information about compensation.***

**Aim 1 Semi-Structured Interview Guide: CLINICIANS**

*Note that this interview is intended to be a guide only – interviewer should ask some or all of these questions depending on what information is shared by participants.*

**Introduction**

**Thank you for your study participation. We want to learn more about your experience using Tele-BE. To learn more about your experience with the new tool that we have developed, we will record this discussion and keep everything you share fully confidential, as allowable by law. Your responses will not be shared with your organization.**

**There are no right or wrong answers; we want to hear your perspectives so we can continue to improve and make our new tool as maximally helpful as possible. You can choose not to answer any questions or can stop the interview at any time.**

**Do you have any questions before we begin?**

**[RECORD]**

1. **Tell me about your experience using the Tele-BE platform.**

***If not discussed, probe:***

- 1. **What did you like about the platform?**
  2. **What did you not like about the platform?**
  3. **Was there anything you expected or wanted to be part of the platform that wasn’t there?**
  4. ***You may have noticed there was a checklist, were there things on the list that you regularly did as part of your session structure that were not included on the checklist? Was there anything here where you would have used different language to describe what you did?***

1. ***Learnability:* How easy or hard was it to navigate Tele-BE?**

***If not discussed, probe:***

- 1. **How, if at all, can we improve how easy it is to learn to use?**
  2. **What training do you think is most important for learning to use Tele-BE?**

1. ***Acceptability* How do you see yourself using something like Tele-BE?**

***If not discussed, probe:***

- 1. **How would it fit or not fit into your practice?**

1. ***Usefulness:* How helpful or unhelpful would it be in supporting your clinical care?**
   1. ***If not discussed, probe:* In what ways, if at all, would it change your clinical care?**
2. ***Satisfaction with Aesthetics:* Tell me about your overall opinions about the look of Tele-BE.**

***If not discussed, probe:***

- 1. ***Probe specific features if not discussed:***
     1. ***Color***
     2. ***Font***
     3. ***Arrangement***
  2. **What, if any, areas do you think could be improved?**

1. ***Efficiency & Workflow Integration:* How can Tele-BE best be incorporated into your clinical workflow?**
   1. ***If not discussed, probe:* Do you see any barriers to incorporating it into your workflows?**

**Thank you so much for your time. We really appreciate all of this feedback. Is there anything else you would like to share with us about other suggestions or improvements?**

***Provide information about compensation.***
