# Supplementary material for: Behavioral Nudges to Enhance Fidelity in Telehealth Sessions (BENEFITS): Protocol for Developing and Pilot Testing a Telehealth Tool to Improve Cognitive Behavioral Therapy Implementation
Source: JMIR Res Protoc. 2025 Sep 18;14:e76035. doi: 10.2196/76035 (PMC12491885; doi:10.2196/76035)
Supplement: Multimedia Appendix 3 [file resprot_v14i1e76035_app3.docx]

**Aim 2 Semi-Structured Interview Guide: CLINICIANS**

*Note that this interview is intended to be a guide only – interviewer should ask some or all of these questions depending on what information is shared by participants.*

**Introduction**

**Thank you for your study participation. We want to learn more about your experience using Tele-BE. To learn more about your experience with the new tool that we have developed, we will record this discussion and keep everything you share fully confidential, as allowable by law. Your responses will not be shared with your organization.**

**There are no right or wrong answers; we want to hear your perspectives so we can continue to improve and make our new tool as maximally helpful as possible. You can choose not to answer any questions or can stop the interview at any time.**

**Do you have any questions before we begin?**

**[RECORD]**

1. **Tell me about your experience using the Tele-BE platform.**

***If not discussed, probe:***

- 1. **What did you like about the platform?**
  2. **What did you not like about the platform?**
  3. **Was there anything you expected or wanted to be part of the platform that wasn’t there?**
  4. ***You may have noticed there was a checklist, were there things on the list that you regularly did as part of your session structure that were not included on the checklist? Was there anything here where you would have used different language to describe what you did?***

1. ***Learnability:* How easy or hard was it to navigate Tele-BE?**

***If not discussed, probe:***

- 1. **How, if at all, can we improve how easy it is to learn to use?**
  2. **What training do you think is most important for learning to use Tele-BE?**

1. ***Acceptability* How do you see yourself using something like Tele-BE?**

***If not discussed, probe:***

- 1. **How would it fit or not fit into your practice?**

1. ***Usefulness:* How helpful or unhelpful would it be in supporting your clinical care?**
   1. ***If not discussed, probe:* In what ways, if at all, would it change your clinical care?**
2. ***Satisfaction with Aesthetics:* Tell me about your overall opinions about the look of Tele-BE.**

***If not discussed, probe:***

- 1. ***Probe specific features if not discussed:***
     1. ***Color***
     2. ***Font***
     3. ***Arrangement***
  2. **What, if any, areas do you think could be improved?**

1. ***Efficiency & Workflow Integration:* How can Tele-BE best be incorporated into your clinical workflow?**
   1. ***If not discussed, probe:* Do you see any barriers to incorporating it into your workflows?**

**Thank you so much for your time. We really appreciate all of this feedback. Is there anything else you would like to share with us about other suggestions or improvements?**

***Provide information about compensation.***

***Aim 2:  Semi-Structured Focus Interview Guide: PATIENTS***

*Note that this interview is intended to be a guide only – interviewer should ask some or all of these questions depending on what information is shared by participants.*

***Introduction***

***Thank you for your study participation. We want to learn more about your experience over the past few weeks as your therapist was using the new therapy support tool we developed. The goal of this new tool is to help clinicians structure their sessions to deliver high-quality care to the patients they serve.***

***There are no right or wrong answers; we want to hear your perspectives so we can continue to improve and make our new tool as maximally helpful as possible. You can choose not to answer any questions or you can stop the interview at any time. We will not share your responses with your therapist and your responses will not affect your therapy care plan.***

***Do you have any questions before we begin?***

***[RECORD]***

1. ***First, I’d like to ask about what your therapy sessions have been like, beginning actually by thinking back to before the study started on DATE. To protect your privacy, I’m not asking about what you’ve talked about in therapy. Instead, I’m wondering about the overall experience of your sessions in these past few weeks – for example, whether it felt generally helpful or unhelpful. What was therapy like for you before the study?***
   1. ***probe: Have you noticed any changes in what the sessions were like since the study started? [reassure client if needed that changes or no changes are both okay]***
2. ***Tell me about the quality of your relationship with your therapist overall, before the study started.***
   1. ***If not discussed, probe: Tell me about how connected you felt with your therapist.***
   2. ***Has the quality of your relationship or your connection with your therapist changed at all over the past few weeks? If so, how has it changed?***
3. ***Sometimes in therapy a clinician will open up a session with a collaborative discussion about the goals for that particular session. This is sometimes called the “agenda” or “plan” for the session. Before the study started, did your therapist usually do something like that?***
   1. ***If YES, probe: Tell me more about that and what that is usually like for you.***
      1. ***Did you notice any differences in how your therapist did this over the past few weeks?***
         1. ***Tell me more about the differences you experienced.***
   2. ***If NO, probe: Did you notice any changes related to using an agenda or plan for the session over these last few weeks?***
      1. ***If YES, probe: Tell me more about the changes you noticed.***
4. ***Sometimes in therapy a clinician will work with the client to plan something for them to practice between sessions. This is sometimes called the “homework” or “home practice” for the session. Before the study started, did your therapist usually do something like that?***
   1. ***If YES, probe: Tell me more about what that’s usually like for you***
      1. ***Did you notice any differences in how your therapist did this over the past few weeks?***
         1. ***If YES, probe: Tell me more about the differences you experienced.***
   2. ***If NO, probe: Did you notice any changes related to planning practices or homework over these last few weeks?***
      1. ***If YES, probe: Tell me more about the changes you noticed.***
5. ***Sometimes in therapy a clinician will ask to discuss the previous week’s home practice or homework; this often happens early in a session. Before the study, did your therapist usually do something like that?***
   1. ***If YES, probe: Tell me more about that and what that is usually like for you***
      1. ***Did you notice any differences in how your therapist did this over the past few weeks?***
         1. ***If YES, probe: Tell me more about the differences you experienced.***
   2. ***If NO, probe: Did you notice any changes related to your therapist asking to review homework over these last few weeks?***
      1. ***If YES, probe: Tell me more about the changes you noticed.***
6. ***Sometimes in therapy a clinician will check in about how a client’s mood is at the beginning of the session by asking them to rate their mood on a scale (like 1-10 or 1-100) or by asking clients to fill out brief questionnaires. Before the study, did your therapist usually do something like that?***
   1. ***If YES, probe: Tell me more about that and what that is usually like for you***
      1. ***Did you notice any differences in how your therapist did this over the past few weeks?***
         1. ***If YES, probe: Tell me more about the differences you experienced.***
   2. ***If NO, probe: Did you notice any changes related to your therapist asking about your mood at the beginning of the session over these last few weeks?***
      1. ***If YES, probe: Tell me more about the changes you noticed.***
7. ***Sometimes in therapy a clinician will work to teach a specific strategy or skill to their clients and help them practice it in session. Some examples can be (but are not limited to) relaxation strategies, “catch check change”, activity monitoring, or practicing facing your fears. Does your therapist usually do something like that?***
   1. ***If YES, probe: Tell me more about that and what that is usually like for you***
      1. ***Did you notice any differences in how your therapist did this over the past few weeks?***
         1. ***If YES, probe: Tell me more about the differences you experienced.***
   2. ***If NO, probe: Did you notice any changes related your therapist practicing skills with you over these last few weeks?***
      1. ***If YES, probe: Tell me more about the changes you noticed.***

***Thank you so much for your time. We really appreciate all of this feedback. Is there anything else you would like to share with us about other suggestions or improvements?***

***Provide information about compensation.***
